# Trials and the importance of usual care

**DOI:** 10.1186/s13054-024-04977-1

**Published:** 2024-07-08

**Authors:** Kyle C. White, Kevin B. Laupland, Rinaldo Bellomo, Queensland Critical Care Research Network (QCCRN)

**Affiliations:** 1 Intensive Care Unit, Princess Alexandra Hospital, 199 Ipswich Road, Woolloongabba, Brisbane, QLD 4102 Australia; 2 Intensive Care Unit, Queen Elizabeth II Jubilee Hospital, Coopers Plains, QLD Australia; 3 Faculty of Health, School of Clinical Sciences, Queensland University of Technology, Brisbane, QLD Australia; 4 Mayne Academy of Critical Care, Faculty of Medicine, University of Queensland, St Lucia, QLD Australia; 5 Intensive Care Services, Royal Brisbane and Women’s Hospital, Herston, QLD Australia; 6 Department of Intensive Care, Austin Hospital, Heidelberg, VIC Australia; 7 Australian and New Zealand Intensive Care Research Centre (ANZIC-RC), School of Public Health and Preventive Medicine, Monash University, Melbourne, VIC Australia; 8 Department of Critical Care, University of Melbourne, Melbourne, VIC Australia; 9 Department of Intensive Care, Royal Melbourne Hospital, Melbourne, VIC Australia

Dear Editor,

We read with great interest the REDUSE trial paper by Linden and colleagues [1] and particularly commend the comprehensive protocol that recognised the importance of nutrition to fluid accumulation [2] and detailed instructions on concentrating drug administration.

However, we are concerned about the external validity of fluid input with the usual care arm of the REDUSE trial. Such patients received a median fluid input of 9.76 L in the first three days of ICU stay.

**Fig. 1:**
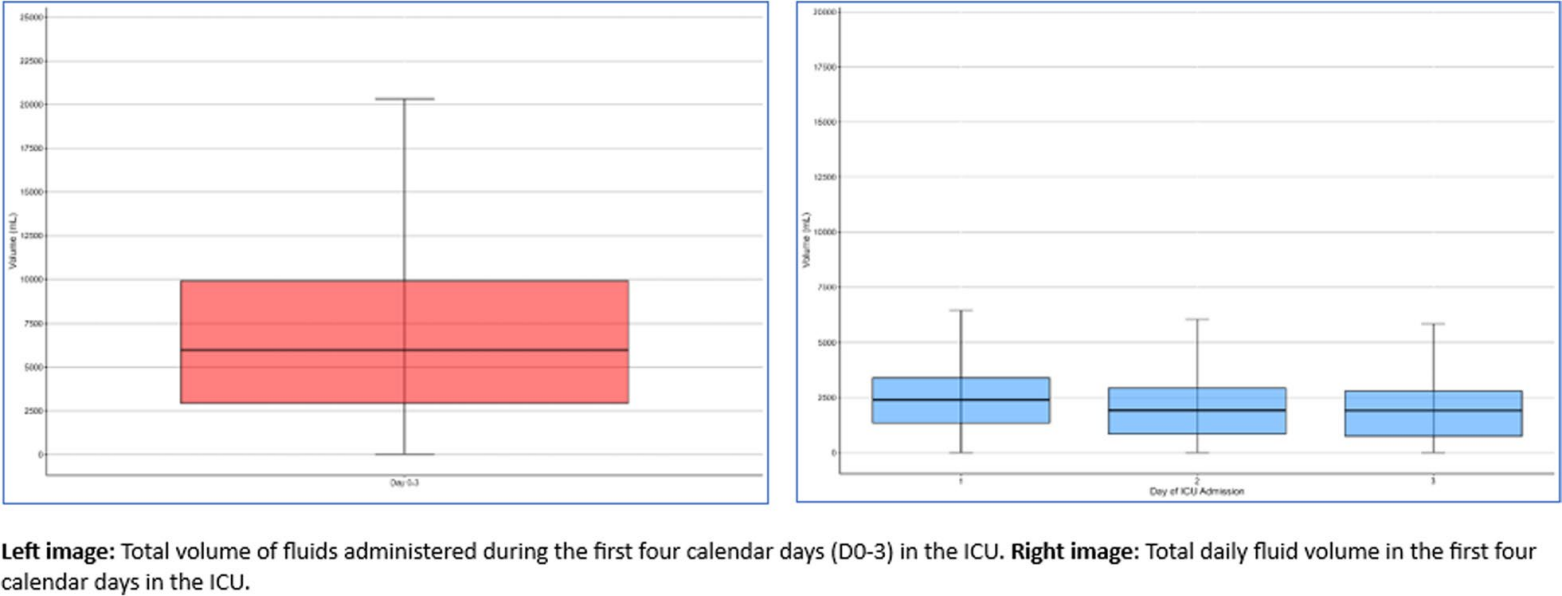
Fluid Administration in patients admitted to ICU with septic shock all sources of fluid input included (crystalloids, colloids, blood products nutrition, and oral sources

In 6412 patients with septic shock, from a previously described cohort [3], admitted to 12 participating ICUs in Australia we found a median fluid input over the first 3 days, D0–D3, of 5.99 L. The overall fluid input over the first three days of ICU admission, together with the single-day breakdown is presented in Fig. 1. The median fluid input of under 6 L was the same as the 6.01 L reported in the intervention arm of the REDUSE trial, demonstrating different baseline practices.

Furthermore, recent evidence in renal replacement therapy has demonstrated profound geographical variation in fluid management practices [4]. The assumption that the results of the trial can applied to different jurisdictions may be inaccurate and could have consequences on future, multinational interventional trials, and, ultimately, patient care.

Second, we would like to stress that the REDUSE trial intervention did not highlight the impact on fluid balance, as this information is relegated to the supplemental material. Recent work in critically ill patients with acute kidney injury has demonstrated the importance of urine output and diuretic therapy to the multi-factor development of fluid accumulation [2]. In the REDUSE trial cumulative fluid balance at day 3 was + 2317 mL in the usual care arm, whereas, in our cohort of > 6000 patients, the median cumulative FB was + 544 mL, D0–D3.

We believe that addressing these concerns will contribute to a more comprehensive understanding of fluid management in critically ill patients and guide future research in this important area.
